# Supplementary material for: A computational platform for high-throughput analysis of RNA sequences and modifications by mass spectrometry
Source: Nat Commun. 2020 Feb 17;11:926. doi: 10.1038/s41467-020-14665-7 (PMC7026122; doi:10.1038/s41467-020-14665-7)
Supplement: Supplementary file 1 — Supplementary Information [file 41467_2020_14665_MOESM1_ESM.pdf]

Wein et al.:

A computational platform for high-throughput analysis of RNA sequences and modifications by mass spectrometry

Supplementary Figures

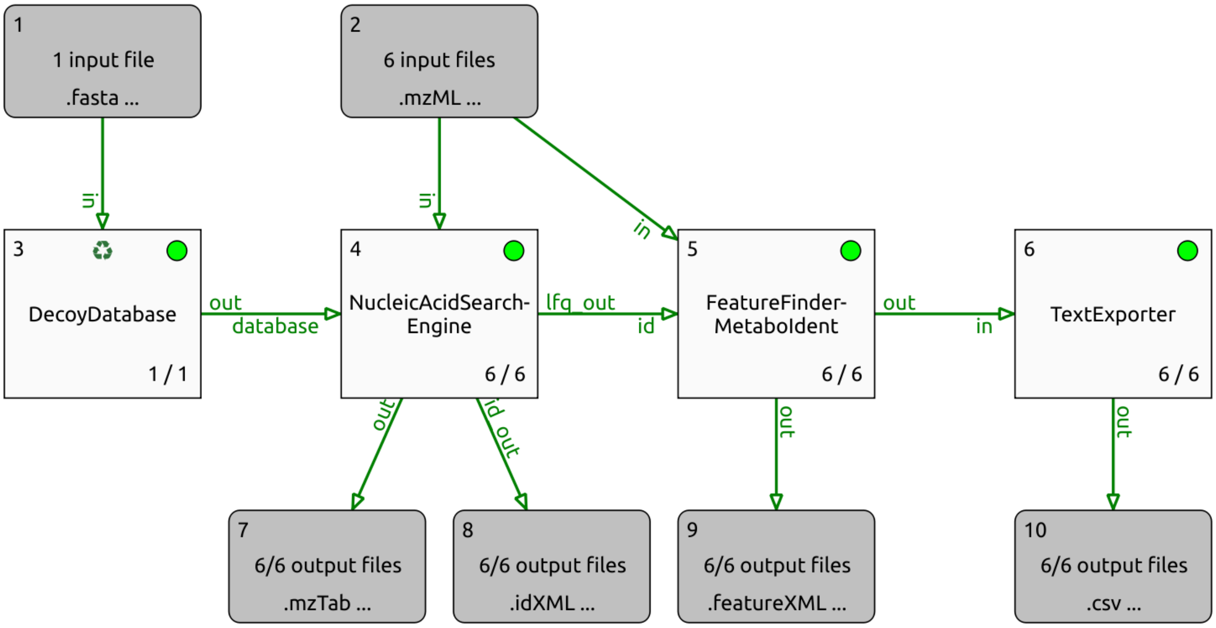

Supplementary Figure 1: Data analysis pipeline for the NME1 data, comprising target/decoy database generation, database search (incl. FDR estimation and filtering), targeted feature detection and data export. Screenshot from TOPPAS, the OpenMS workflow editor. The whole pipeline ran in less than one minute (single-threaded) on our server.

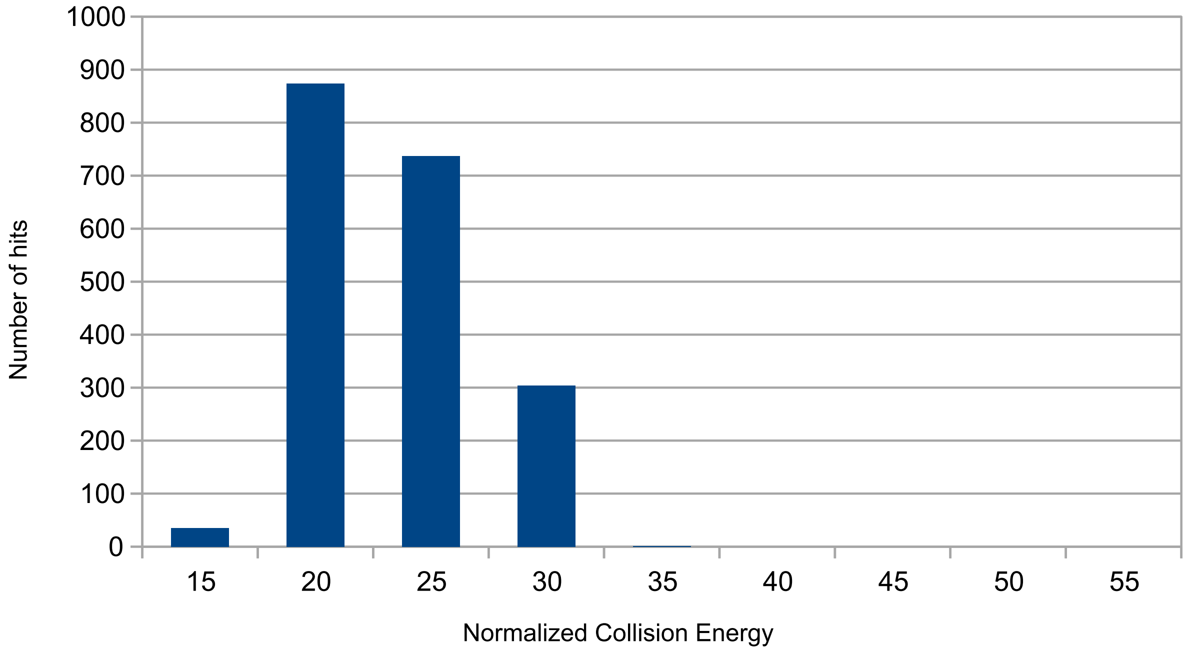

Supplementary Figure 2: Graph showing the relationship between Normalized Collision Energy (NCE) used in HCD and the number of NASE search hits scoring above our cutoff in replicate runs of the let-7 sample. The optimum NCE is 20.

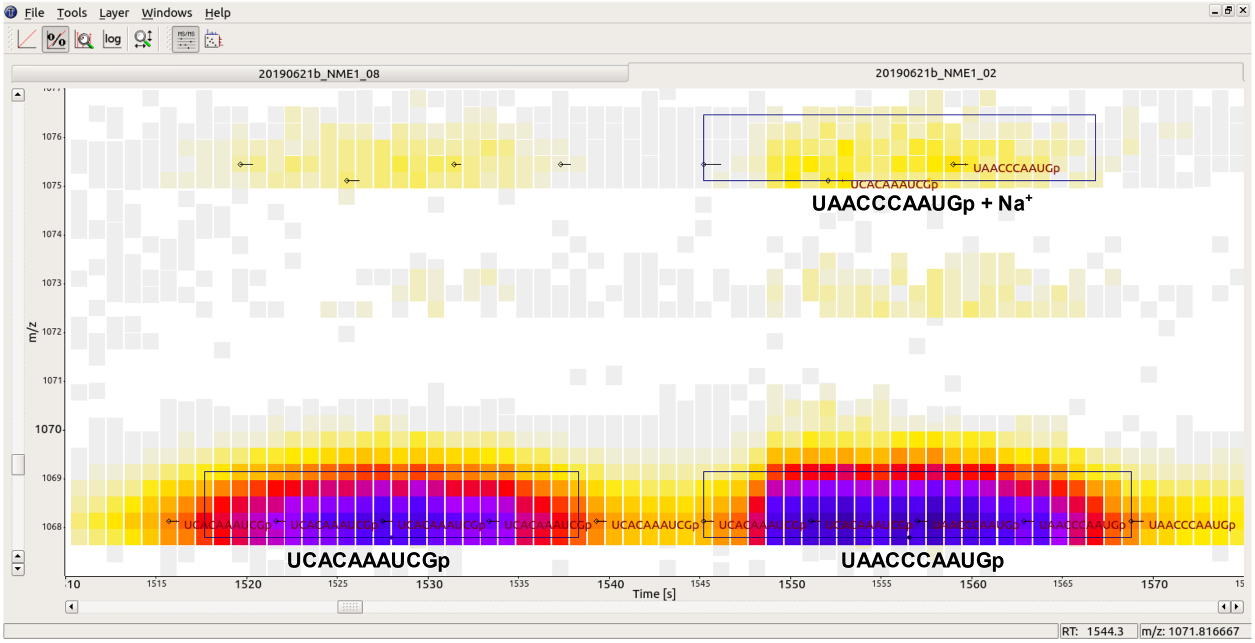

Supplementary Figure 3: Annotated screenshot from TOPPView showing data from the NME1 control sample, corresponding to the NCL1-treated data shown in Fig. 4a. In this example, the peaks for the two isobaric oligonucleotides were better separated chromatographically. Note the loss of signal intensity and sequence identifications for the methylated oligonucleotides, compared to Fig. 4a.

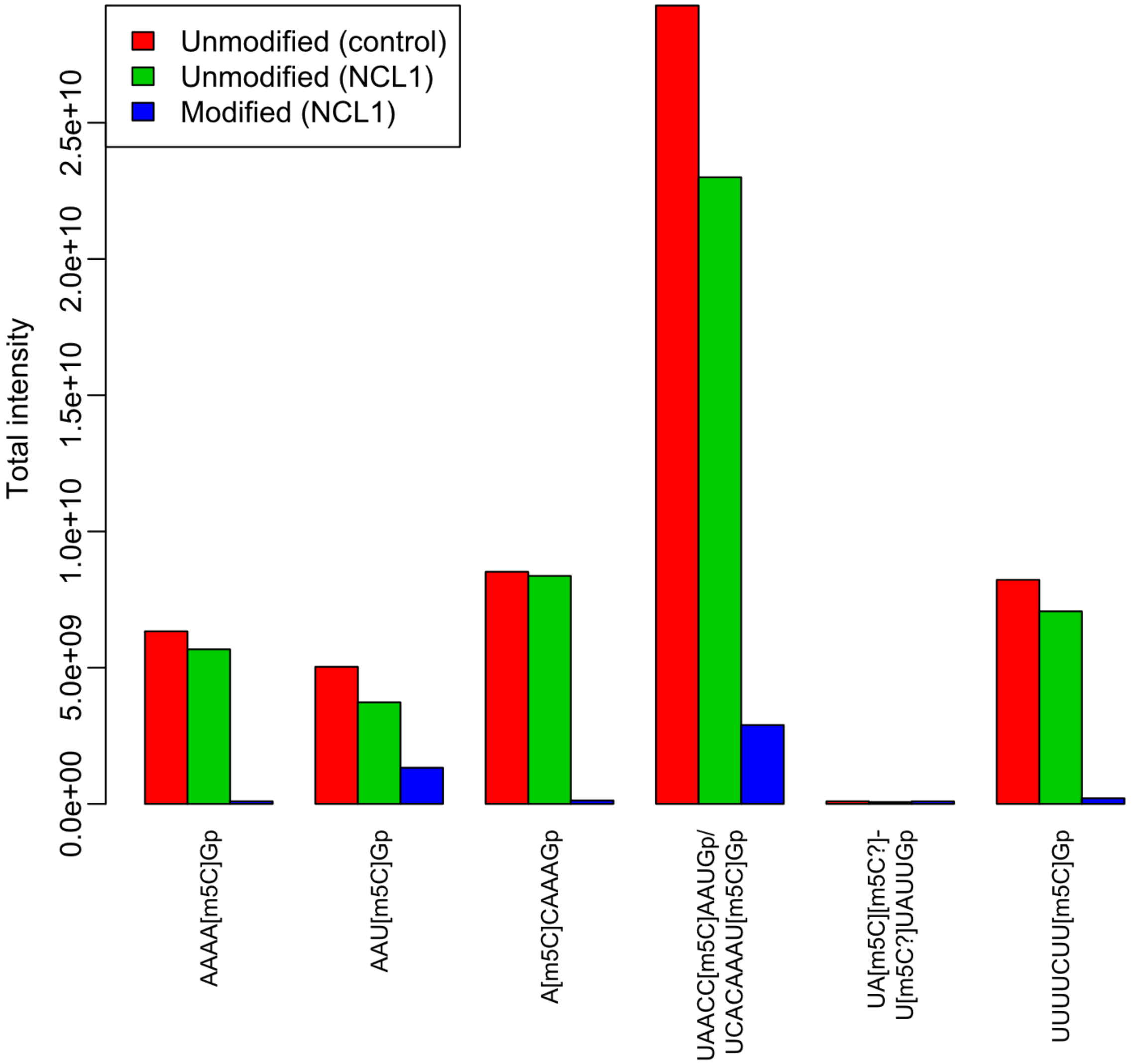

Supplementary Figure 4: Comparison of feature intensities (summed over all charge/adduct variants) for modified oligonucleotides and their unmodified counterparts (e.g. "AAAA[m5C]G" and "AAAACG") in the samples of the NME1 dataset. The intensities of unmodified oligonucleotides in the control are consistently higher than in the NCL1-treated sample, with the difference being roughly proportional to the intensity observed for the modified oligonucleotide.
