# Supplementary material for: A computational platform for high-throughput analysis of RNA sequences and modifications by mass spectrometry
Source: Nat Commun. 2020 Feb 17;11:926. doi: 10.1038/s41467-020-14665-7 (PMC7026122; doi:10.1038/s41467-020-14665-7)
Supplement: Supplementary file 3 — Description of Additional Supplementary Files [file 41467_2020_14665_MOESM3_ESM.pdf]

Description of Additional Supplementary Files

File Name: Supplementary Data 1
Description: Identified oligonucleotides in the human rRNA dataset, compared to published data (Taoka et al., Nucleic Acids Res. 46, 2018). Table columns: 1. rRNA of origin. 2. Oligonucleotide sequence. 3. Number of modifications in the oligonucleotide. 4./5. Start/end position in the rRNA sequence. 6. Number of spectral counts in the filtered result set. 7. Status compared to the published data, color-coded according to the level of correctness; either “correct” (green), “modification ambiguously localized”/“modification mis-localized” (yellow), or “modification missed”/“spurious modification”/“wrong modification” (red). 8. Expected modifications in this oligonucleotide according to the published data. 9. Comments.
