# Supplementary material for: A computational platform for high-throughput analysis of RNA sequences and modifications by mass spectrometry
Source: Nat Commun. 2020 Feb 17;11:926. doi: 10.1038/s41467-020-14665-7 (PMC7026122; doi:10.1038/s41467-020-14665-7)
Supplement: Supplementary file 5 — Reporting Summary [file 41467_2020_14665_MOESM5_ESM.pdf]

nature research

Corresponding author(s): Hendrik Weisser

Last updated by author(s): Jan 8, 2020

Reporting Summary

Nature Research wishes to improve the reproducibility of the work that we publish. This form provides structure for consistency and transparency in reporting. For further information on Nature Research policies, see Authors & Referees and the Editorial Policy Checklist.

Statistics

For all statistical analyses, confirm that the following items are present in the figure legend, table legend, main text, or Methods section.

n/a	Confirmed	
☒	☐	The exact sample size (*n*) for each experimental group/condition, given as a discrete number and unit of measurement
☒	☐	A statement on whether measurements were taken from distinct samples or whether the same sample was measured repeatedly
☒	☐	The statistical test(s) used AND whether they are one- or two-sided<br>*Only common tests should be described solely by name; describe more complex techniques in the Methods section.*
☒	☐	A description of all covariates tested
☒	☐	A description of any assumptions or corrections, such as tests of normality and adjustment for multiple comparisons
☒	☐	A full description of the statistical parameters including central tendency (e.g. means) or other basic estimates (e.g. regression coefficient) AND variation (e.g. standard deviation) or associated estimates of uncertainty (e.g. confidence intervals)
☒	☐	For null hypothesis testing, the test statistic (e.g. *F*, *t*, *r*) with confidence intervals, effect sizes, degrees of freedom and *P* value noted<br>*Give P values as exact values whenever suitable.*
☒	☐	For Bayesian analysis, information on the choice of priors and Markov chain Monte Carlo settings
☒	☐	For hierarchical and complex designs, identification of the appropriate level for tests and full reporting of outcomes
☒	☐	Estimates of effect sizes (e.g. Cohen's *d*, Pearson's *r*), indicating how they were calculated

Our web collection on statistics for biologists contains articles on many of the points above.

Software and code

Policy information about availability of computer code

Data collection	Thermo Fisher Scientific's Xcalibur 4.0 and Exactive Series 2.8 software were used for mass spectrometry (MS) data acquisition. ProteoWizard 3.0.10875 was used for data conversion.
Data analysis	MS data analysis was performed with a development version of OpenMS 2.4, in particular with the newly developed NucleicAcidSearchEngine. Further data analysis and visualization was performed with R 3.5.1. Adobe Illustrator, Inkscape and LibreOffice Calc were used in the creation of figures.

For manuscripts utilizing custom algorithms or software that are central to the research but not yet described in published literature, software must be made available to editors/reviewers. We strongly encourage code deposition in a community repository (e.g. GitHub). See the Nature Research guidelines for submitting code & software for further information.

Data

Policy information about availability of data

All manuscripts must include a data availability statement. This statement should provide the following information, where applicable:

- Accession codes, unique identifiers, or web links for publicly available datasets
- A list of figures that have associated raw data
- A description of any restrictions on data availability

Mass spectrometry data files and search results (as well as label-free quantification results for the “NME1” dataset) were deposited in the PRIDE repository (https://www.ebi.ac.uk/pride/archive/) with dataset identifiers PXD012094 (synthetic let-7), PXD016308 (NME1), PXD016323 (human rRNA) and PXD016328 (human tRNA).

Field-specific reporting

Please select the one below that is the best fit for your research. If you are not sure, read the appropriate sections before making your selection.

☒ Life sciences ☐ Behavioural & social sciences ☐ Ecological, evolutionary & environmental sciences

For a reference copy of the document with all sections, see nature.com/documents/nr-reporting-summary-flat.pdf

Life sciences study design

All studies must disclose on these points even when the disclosure is negative.

Sample size	Four datasets containing one to three biological samples were analyzed. This was sufficient to validate the analysis software.
Data exclusions	No data was excluded from analyses, but results were filtered according to dataset-specific confidence criteria (see Results section).
Replication	No replication was attempted
Randomization	There were no experimental groups
Blinding	There was no group allocation; blinding was not relevant.

Reporting for specific materials, systems and methods

We require information from authors about some types of materials, experimental systems and methods used in many studies. Here, indicate whether each material, system or method listed is relevant to your study. If you are not sure if a list item applies to your research, read the appropriate section before selecting a response.

Materials & experimental systems

n/a	Involved in the study	
☒	☐	Antibodies
☐	☒	Eukaryotic cell lines
☒	☐	Palaeontology
☒	☐	Animals and other organisms
☒	☐	Human research participants
☒	☐	Clinical data

Methods

n/a	Involved in the study	
☒	☐	ChIP-seq
☒	☐	Flow cytometry
☒	☐	MRI-based neuroimaging

Eukaryotic cell lines

Policy information about cell lines

Cell line source(s)	HAP1 cells from Horizon Discovery (Cambridge, UK)
Authentication	Cell line was not authenticated
Mycoplasma contamination	Cell line was not tested for mycoplasma contamination
Commonly misidentified lines (See ICLAC register)	*Name any commonly misidentified cell lines used in the study and provide a rationale for their use.*
